# Beyond Disposability: A Systematic Review of Reusable and Single-Use Products in Hygiene-Related Nursing Care in the ICU

**DOI:** 10.3390/nursrep16090335

**Published:** 2026-09-13

**Authors:** Eleonora Lombardi, Gianluca Barra, Davide Vicari, Luciano Midolo, Floriana Pinto, Francesco Limonti, Roberta Decaro, Francesca Trotta, Davide Bartoli

**Affiliations:** 1 Italian National Association of Critical Care Nurses (ANIARTI), Via Francesco Nullo 6/A, 16167 Genoa, Italy; 2 Department of Biomedicine and Prevention, University of Rome Tor Vergata, Via Montpellier 1, 00133 Rome, Italy; 3 Department of Health Professions, Sant’Andrea University Hospital, Via di Grottarossa 1035, 00189 Rome, Italy; 4 Department of Wellbeing, Health and Environmental Sustainability (BeSSA), Sapienza University of Rome, 02100 Rieti, Italy

**Keywords:** intensive care units, nursing care, patient hygiene, reusable products, single-use products, environmental sustainability, healthcare costs, life cycle assessment

## Abstract

**Background/Objectives:** Intensive care is resource-intensive, and routine nursing hygiene contributes substantially to material consumption. This systematic review synthesised evidence comparing single-use and reusable or traditional reusable products in hygiene-related nursing care in intensive and critical care settings, considering clinical, economic, and environmental outcomes. **Methods:** A systematic review with structured narrative synthesis was conducted in accordance with PRISMA 2020. MEDLINE via PubMed, Scopus, Web of Science Core Collection, and CINAHL Ultimate via EBSCOhost were searched from inception to 25 August 2026. No language, date, or publication-type filters were applied at the database level. Primary empirical comparative studies and process-based life cycle assessments reporting original inventory data were eligible; English or Italian full text was required for inclusion. Two reviewers independently screened studies, extracted data, and performed design-appropriate methodological appraisal, with a third reviewer available to resolve disagreements. The review was registered in PROSPERO (CRD420261376160). **Results:** Eight studies were included: four quasi-experimental or before–after studies, three randomized trials, and one process-based life cycle assessment. The seven patient-based studies included 2665 participants across adult, paediatric, and neonatal intensive care settings. Environmental evidence was limited to two complementary studies from a single Australian ICU: reusable linen was associated with a 50% reduction in carbon emissions, from 7206 to 3605 kg CO_2_e, and substantial reductions in landfill waste. Economic findings were product- and context-dependent; disposable bathing systems and diapers were generally less costly in the settings examined, whereas reusable linen increased overall expenditure by approximately 3%. Clinical findings showed no uniform advantage for either product category. After adjustment, linen type was not independently associated with pressure injury, bathing studies showed heterogeneous microbiological findings and no consistent physiological advantage between methods, and one neonatal before–after study reported lower probable sepsis with disposable diapers. **Conclusions:** Current evidence does not support a general advantage of either reusable or single-use hygiene products across intensive care. Environmental benefits were demonstrated for reusable linen, whereas economic and clinical outcomes varied by product, setting, and analytical approach. The evidence base remains small and heterogeneous, and no included study assessed clinical, economic, and environmental outcomes concurrently. Integrated comparative studies, particularly in European intensive care settings, are needed.

## 1. Introduction

Intensive care units (ICUs) are among the most resource-intensive environments in modern healthcare [1]. Each patient-day generates approximately 7.1 kg of waste and carbon emissions ranging between 88 and 178 kg CO_2_e, with consumables and single-use disposable products representing a major contributor to this burden [2,3]. A substantial but largely unquantified fraction of this consumption is driven by hygiene-related nursing care, bed bathing, oral care, skin cleansing, and perineal hygiene, procedures performed daily on patients who are almost entirely dependent on nursing staff for all activities of daily living [4].

Beyond their environmental footprint, hygiene-related nursing interventions have important implications for skin integrity, microbial control, patient comfort, and infection prevention [5,6]. Although these practices have been increasingly evaluated in ICU populations, the evidence remains heterogeneous; no consensus on optimal protocols has emerged, and substantial variability persists across institutions [7,8].

Within this context, the resource implications of ICU hygiene practice have attracted growing attention: the reliance on single-use disposable products generates substantial and inconsistently measured material consumption and healthcare waste, while reusable alternatives proposed across several hygiene domains remain poorly characterized in terms of clinical equivalence, economic costs, and environmental impact [9,10]. Comparative life-cycle analyses of reusable versus disposable healthcare textiles suggest that reusable systems may substantially reduce greenhouse gas emissions and solid waste generation, although findings remain context-dependent and influenced by laundering and transport processes [10].

Despite growing interest in sustainable ICU practice, evidence regarding hygiene-related nursing care remains fragmented across products, interventions, and clinical settings [9]. Existing studies predominantly focus on individual products, devices, or infection-prevention strategies rather than examining hygiene-related nursing care as an integrated practice domain [11]. Examples include chlorhexidine-based cleansing products and oral care devices [12,13], as well as healthcare textiles and personal protective equipment [14]. Furthermore, different outcome domains are typically evaluated separately: infection-control recommendations primarily emphasize patient safety and the prevention of healthcare-associated infections [15], sustainability initiatives focus on environmental impacts and resource stewardship [16], and economic evaluations mainly examine healthcare costs and resource utilization [17]. Consequently, the comparative implications of reusable and disposable products across clinical, economic, environmental, and infection-related domains remain poorly understood, and only limited literature has adopted an integrated perspective that simultaneously considers these dimensions in ICU hygiene practice [9]. Given the extensive reliance of routine ICU care on single-use materials and resource-intensive processes, this knowledge gap has important implications for healthcare sustainability and resource stewardship [18].

This systematic review aims to address this gap by synthesising the available evidence on hygiene-related nursing interventions in intensive care settings as an integrated practice domain, with specific attention to the clinical outcomes, economic costs, and environmental implications of disposable versus reusable products. To our knowledge, no previous systematic review has synthesised the clinical, economic, and environmental dimensions of hygiene-related nursing care in intensive care settings within a single analytical framework.

## 2. Materials and Methods

This systematic review was conducted in accordance with the Preferred Reporting Items for Systematic Reviews and Meta-Analyses (PRISMA) 2020 statement [19]. The review was registered in the International Prospective Register of Systematic Reviews (PROSPERO; CRD420261376160) on 12 May 2026. The final database searches were conducted after registration and before the definitive study-selection and evidence-synthesis procedures reported in this review.

### 2.1. Clinical Question and PICOS Framework

To formulate a specific and systematically structured clinical question, the PICOS framework [20] was adopted:

(P) Population. Patients admitted to intensive care units (ICUs) or critical care settings, of any age group, regardless of admission diagnosis or length of stay.

(I) Intervention. Nursing hygiene practices performed in ICUs or critical care settings, including bed bathing (washcloth bath, no-rinse bath, chlorhexidine-impregnated cloths), oral hygiene, skin cleansing, perineal care, and diapering, using either disposable or reusable products.

(C) Comparator. Disposable (single-use) products versus reusable products or traditional reusable systems.

(O) Outcomes. Environmental outcomes (waste generation, CO_2_-equivalent emissions, life cycle assessment results), economic outcomes (cost per patient per procedure, cost per patient-day, total cost), and clinical outcomes (incidence of healthcare-associated infections, impairment of skin integrity, microbial load). Secondary outcomes included patient comfort and nursing workload.

(S) Study design. Eligible designs comprised primary empirical studies, including randomized controlled trials (RCTs), randomized crossover trials, quasi-experimental and before-and-after studies, prospective and retrospective cohort studies, and process-based life cycle assessment (LCA) studies reporting original inventory data. Systematic and narrative reviews, text and opinion papers, editorials, letters, conference abstracts, and other non-primary publications were excluded.

Based on this framework, the review question of the present review is as follows:

What are the environmental and economic impacts, and what clinical outcomes are associated with the use of disposable versus reusable products in nursing hygiene practices in intensive care units and critical care settings?

### 2.2. Search Strategy and Data Sources

**Figure 1:**
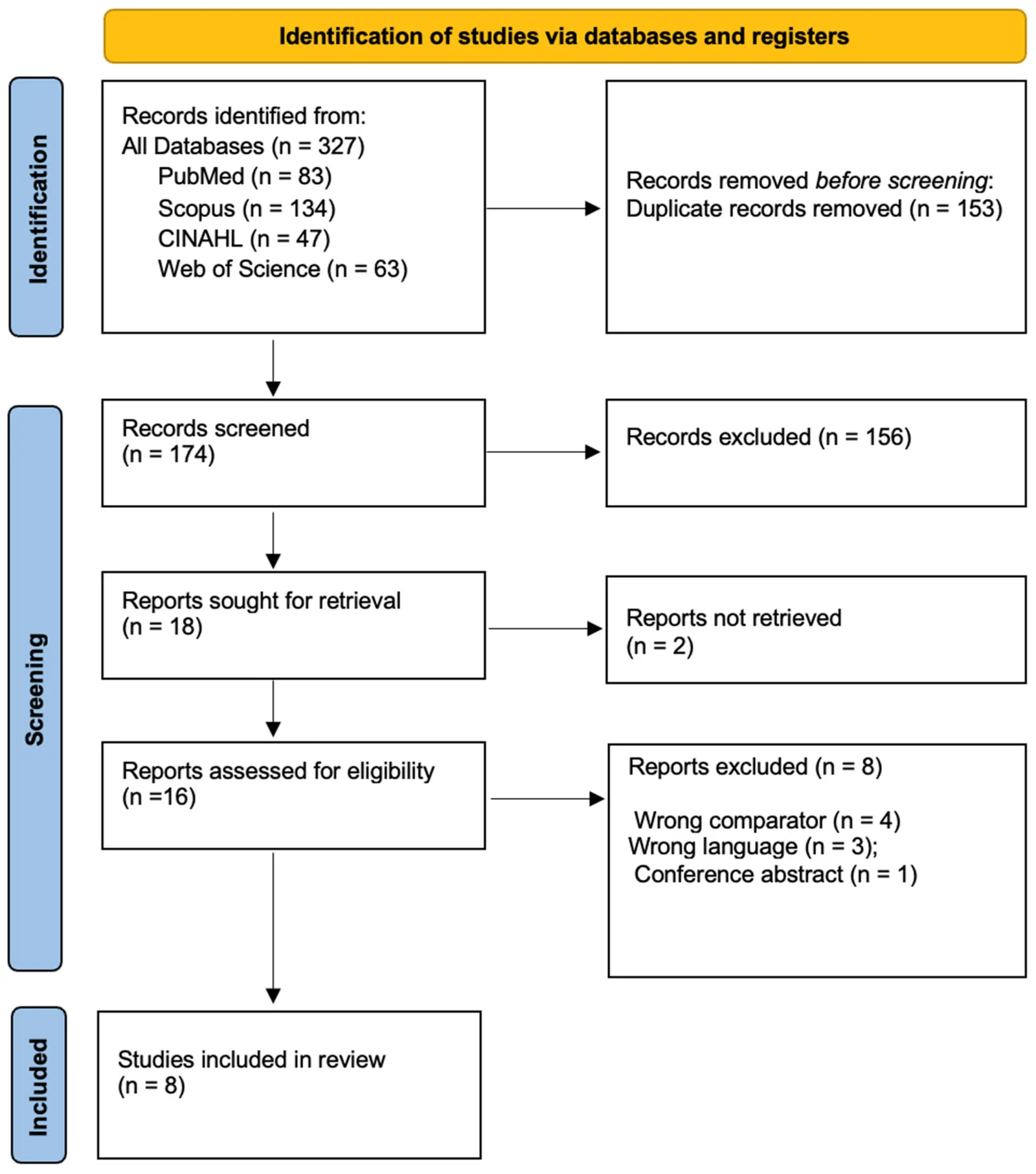
PRISMA 2020 Flow Diagram of the study selection process [19]. This work is licensed under CC BY 4.0. To view a copy of this license, visit https://creativecommons.org/licenses/by/4.0/ (accessed on 6 September 2026).

The electronic searches of MEDLINE via PubMed, Scopus, Web of Science Core Collection, and CINAHL Ultimate via EBSCOhost were conducted on 25 August 2026. All databases were searched from inception, with no restrictions on publication date. All records reported in the PRISMA 2020 flow diagram (Figure 1) derive from these searches.

The search strategy was informed by the PICOS framework and organized into three concept blocks combined with the Boolean operator AND: (i) population and setting, including intensive and critical care; (ii) hygiene-related products and practices; and (iii) product type and comparator, including disposable, single-use, and reusable products. Within each block, database-specific controlled vocabulary was combined with free-text terms using OR, including Medical Subject Headings (MeSH) in MEDLINE via PubMed and CINAHL Headings in CINAHL. Scopus and Web of Science, which do not use a comparable controlled vocabulary for this purpose, were searched using corresponding free-text terms in the title, abstract, and keyword fields. The MEDLINE via PubMed strategy is reported in Table 1, and the complete database-specific line-by-line search histories, including the exact search syntax, number of records retrieved at each step, and date of execution, are provided in Supplementary Table S1. No language, date, or publication-type filters were applied at the database level; all eligibility criteria, including the language criterion, were applied during screening and full-text assessment, and reports excluded on the basis of language are documented in the PRISMA 2020 flow diagram. The search was limited to records indexed in the four selected bibliographic databases to maintain a transparent, reproducible, and consistent retrieval process across sources. Grey literature was not searched, and neither backward reference checking nor forward citation searching was undertaken.

### 2.3. Eligibility Criteria

The following inclusion and exclusion criteria were applied for the purposes of this review.

Inclusion criteria: (a) primary empirical studies (randomized controlled trials, including crossover trials, quasi-experimental and before-and-after studies, and cohort studies) and process-based life cycle assessments reporting original inventory data, with no restriction on publication date; (b) studies conducted on patients admitted to ICUs or critical care settings, including neonatal and pediatric intensive care, regardless of age group; (c) studies evaluating disposable, single-use, or reusable products in nursing hygiene practices and reporting at least one of the predefined outcomes (clinical, economic, or environmental); and (d) articles published in English or Italian. The language restriction was prespecified in the PROSPERO protocol and reflected the language competencies of the review team; the number of reports excluded on this basis at the full-text stage is reported in the PRISMA 2020 flow diagram (Figure 1).

Exclusion criteria: (a) studies not directly comparing a disposable or single-use hygiene product with a traditional or reusable alternative; (b) narrative reviews, systematic reviews, text and opinion papers, editorials, letters, commentaries, and conference abstracts; (c) case reports and case studies; (d) studies published in a language other than English or Italian; (e) ongoing studies or protocols without complete outcome data; and (f) reports for which the full text could not be obtained were classified as not retrieved rather than excluded (see Section 2.4).

### 2.4. Study Selection Process

The study selection process was conducted using Rayyan systematic review software (https://www.rayyan.ai/, accessed on 6 September 2026) [21]. Screening, assessment, and study inclusion were independently performed by two reviewers (DV and GB), with a third reviewer (EL) involved in cases of disagreement. The BLIND-ON mode was activated to allow reviewers to work independently and simultaneously in a blinded manner. Titles and abstracts were screened against the predefined eligibility criteria, and the full texts of all potentially relevant records were then sought and assessed. Reports whose full text could not be obtained, after searching the publisher platform and institutional holdings and contacting the corresponding author, were classified as reports not retrieved and are reported as such in the PRISMA 2020 flow diagram; they were not counted among the reports excluded at the full-text stage. Disagreements were resolved through discussion until consensus was reached. A single primary reason for exclusion was recorded for every report excluded at the full-text stage, in accordance with the PRISMA 2020 statement [19].

### 2.5. Data Extraction

Data were independently extracted from each included study by two reviewers (DV and GB) using a standardized data extraction form developed a priori and pilot-tested on five studies not included in the review to assess its clarity and completeness. Disagreements were resolved through discussion and, when necessary, with the involvement of a third reviewer (EL). For each included study, the following data were extracted: (i) study characteristics, including first author, year of publication, country, study design, and setting; (ii) population characteristics, including sample size, age group, and admission diagnosis; (iii) intervention characteristics, including the type of hygiene practice, product description (disposable versus reusable), and any active agent used; and (iv) environmental, economic, and clinical outcomes, including all reported time points. For the life cycle assessment, the extraction form additionally captured the functional unit, the system boundary, the sources and representativeness of the inventory data, the allocation approach, the impact assessment method, and any scenario or sensitivity analyses reported.

### 2.6. Methodological Quality Assessment

The methodological quality of the included studies was assessed using design-appropriate appraisal instruments. Joanna Briggs Institute (JBI) critical appraisal tools were applied to the seven empirical studies [22]: the JBI Critical Appraisal Checklist for Randomized Controlled Trials (13 items) was applied to the three randomized trials, and the revised JBI Critical Appraisal Checklist for Quasi-Experimental Studies (9 items) to the four quasi-experimental and before–after studies. No other JBI checklist was used, because the final set of included studies contained no analytical cross-sectional study and no text or opinion paper. Appraisal was conducted independently by two reviewers (DV and GB), with each reviewer blinded to the other reviewer’s ratings until completion of the initial appraisal. A third reviewer (EL) was consulted in cases of disagreement.

The process-based life cycle assessment was not appraised using a JBI clinical-study checklist, as such instruments were not considered appropriate for this design. Instead, it was assessed against criteria derived from ISO 14040 [23] and ISO 14044 [24] and the LCA transparency framework proposed by Keil et al. [25]. These criteria addressed goal and scope definition, the functional unit, system boundaries, sources and representativeness of inventory data, allocation procedures, the impact assessment method, reporting of greenhouse gases, scenario and sensitivity analyses, formal data-quality and completeness assessment, and independent external critical review. Because these criteria primarily assess methodological transparency and are not directly commensurable with the JBI checklists, the LCA appraisal was reported descriptively, item by item, without conversion to a numerical or percentage score.

For the JBI assessments, each item was rated as “Yes”, “No”, “Unclear”, or “Not applicable”. Item-level judgements are presented in Section 3.2 and were not converted into summary percentage scores, because such scores assign equal weight to checklist items and do not represent a validated summary measure of methodological quality or risk of bias. No percentage threshold or quality cut-off was applied, and no study was excluded on the basis of methodological appraisal [22]. Instead, identified methodological limitations were carried forward into the narrative synthesis and used to inform the interpretation of individual findings. The separate appraisal of the life cycle assessment is reported descriptively in Section 3. 

### 2.7. Assessment of Reporting Biases

Publication bias and small-study effects were not formally assessed because the evidence base was too small and heterogeneous to support a meaningful interpretation of funnel plots or regression-based tests. Selective outcome reporting could not be assessed systematically because protocols or prespecified analysis plans were unavailable for most included studies.

### 2.8. Certainty of the Evidence

A formal GRADE assessment was not undertaken. The review addressed distinct clinical, economic, and environmental questions, and the evidence was synthesised narratively rather than through outcome-specific pooled estimates. The JBI critical appraisal tools, together with the ISO 14040/14044-based transparency appraisal applied to the life cycle assessment, were used to inform the interpretation of individual studies and the narrative synthesis. These assessments should not be interpreted as a formal judgement of certainty for the overall body of evidence [26,27].

### 2.9. Data Synthesis

Due to the substantial methodological and clinical heterogeneity of the included studies, encompassing different study designs (randomized controlled trials, quasi-experimental and before–after studies, and one process-based life cycle assessment), different hygiene products and comparators, heterogeneous outcomes (clinical, economic, and environmental), and different care settings (adult, pediatric, and neonatal intensive care), no quantitative meta-analysis was performed. Instead, data were synthesised through a structured narrative synthesis conducted in accordance with the guidance proposed by Popay et al. [28] and organised by outcome domain rather than study by study.

This approach involved four stages: the development of a preliminary theoretical framework regarding the effects of the intervention; the generation of a preliminary synthesis through tabulation and thematic grouping of studies according to outcome domains (environmental, economic, and clinical); the exploration of relationships within the data, focusing on the direction, magnitude, and consistency of the reported effects across studies; and the assessment of the robustness of the synthesis, taking into account the methodological quality of the included studies.

The findings were presented in a structured data extraction table and discussed narratively for each outcome domain, highlighting areas of convergence and uncertainty across the available evidence.

## 3. Results

### 3.1. Study Selection

The updated database searches conducted on 25 August 2026 identified 327 records: MEDLINE via PubMed (*n* = 83), Scopus (*n* = 134), Web of Science Core Collection (*n* = 63), and CINAHL Ultimate via EBSCOhost (*n* = 47). After removal of 153 duplicate records, 174 records were screened by title and abstract, of which 156 were excluded because they did not meet the predefined eligibility criteria. The full texts of 18 potentially eligible reports were sought for retrieval; 2 reports could not be obtained and were classified as reports not retrieved. Sixteen reports were therefore assessed for eligibility in full text. Of these, 8 were excluded for the following primary reasons: wrong comparator (*n* = 4), language other than English or Italian (*n* = 3), and wrong publication type (conference abstract; *n* = 1). Eight studies met the eligibility criteria and were included in the review and narrative synthesis. The study selection process is summarised in the PRISMA 2020 flow diagram (Figure 1) [19].

### 3.2. Results of the Methodological Quality Assessment

Four quasi-experimental or before–after studies were assessed using the revised JBI Critical Appraisal Checklist for Quasi-Experimental Studies [29,30,31,32]. Overall, the studies met most checklist criteria, although specific methodological limitations were identified. Feely et al. [31] showed limitations in group comparability, with significant differences in APACHE III score and ICU length of stay between study periods, and did not include multiple outcome measurements before and after the intervention. Babu et al. [30] also lacked multiple pre- and post-intervention measurements. In Larson et al. [32], comparability of concomitant care across the two bathing days was unclear. However, the within-patient design was analysed using paired methods appropriate to the available data; accordingly, Q4 was rated “Unclear” and Q9 “Yes”. Follow-up completeness was also not adequately described. In Amir et al. [29], Q9 was rated “Unclear” because the statistical approach described in the Methods section of the original study was not fully consistent with the tests reported in the Results section. Item-level judgements are reported in Table 2.

Three randomized trials were assessed using the JBI Critical Appraisal Checklist for Randomized Controlled Trials [33,34,35]. Allocation concealment was unclear in Tai et al. [35] and Ayhan and Yılmaz [33], and was not adequately reported in Öz et al. [34]. Blinding of those delivering the intervention was not achieved across the three trials, while outcome-assessor blinding was absent in Tai et al. [35] and Ayhan and Yılmaz [33] and unclear in Öz et al. [34]. In Öz et al. [34], true randomization was nevertheless supported by a dice-roll allocation procedure; the principal concerns were the absence of allocation concealment and a significant baseline age difference between groups. For the crossover trial by Ayhan and Yılmaz [33], the appropriateness of crossover-specific design safeguards was rated as unclear. Item-level judgements are reported in Table 3.

The process-based life cycle assessment by Feely et al. [13] was appraised separately using criteria informed by ISO 14040 and ISO 14044 and the methodological transparency framework used by Keil et al. [25]. The study showed high methodological transparency across the principal LCA domains, including the study aim, functional unit, system boundary, inventory data sources, allocation procedures, impact assessment method, product-level results, scenario and sensitivity analyses, and limitations. The main transparency limitations were the absence of a formal data-completeness or data-quality assessment, the lack of an explicit list of the individual greenhouse gases contributing to CO_2_-equivalent estimates, and the absence of a reported independent external critical review. The full appraisal is presented in Table 4.

### 3.3. Characteristics of Included Studies

The eight included studies were heterogeneous in terms of study design, setting, and type of hygiene product evaluated. With regard to study design, the following were represented: four quasi-experimental or before–after studies [29,30,31,32], three randomized trials [33,34,35], and one process-based life cycle assessment [13].

Among the seven patient-based studies, a total of 2665 participants were included: 2114 patients from a metropolitan ICU in Australia [31], 253 neonates from a level II NICU in India [30], 22 patients from a general surgery ICU in Turkey [33], 30 adult ICU patients in Indonesia [29], 138 critically ill patients in Taiwan [35], 68 children in a pediatric ICU in Turkey [34], and 40 patients from medical, surgical, and cardiothoracic ICUs in the United States [32]. The process-based life cycle assessment [13] did not contribute an independent patient population.

Geographically, two studies were conducted in Australia [13,31], two in Turkey [33,34], and one each in Indonesia [29], Taiwan [35], the United States [32], and India [30]. Care settings included adult ICUs, a pediatric intensive care unit, and a neonatal intensive care unit.

The hygiene products evaluated included reusable versus disposable bed linen and underpads [13,31], disposable or dry bed-bathing systems versus traditional basin or soap-and-water bathing [29,32,33,34,35], and disposable versus reusable cloth diapers [30]. The full characteristics of the included studies and extracted data are presented in Table 5.

### 3.4. Environmental Outcomes

Two of the eight included studies reported direct environmental outcomes, and only one did so through a formal life cycle assessment [13,31]. Ayhan and Yılmaz [33] additionally reported differences in consumable use, providing an indirect indicator of material resource use rather than a formal environmental outcome. The remaining five studies [29,30,32,34,35] did not assess environmental impact. The process-based life cycle assessment by Feely et al. [13] provided the only quantified life-cycle estimate of greenhouse gas emissions: substituting reusable underpads and bed protectors for their disposable equivalents in one metropolitan ICU reduced total carbon emissions from 7206 to 3605 kg CO_2_e, corresponding to approximately 3.6 tonnes of CO_2_e avoided, together with approximately 2.2 tonnes of landfill waste avoided. The accompanying before–after study by Feely et al. [31] reported the elimination of approximately 496 kg of landfill waste per year in the same unit. Because both reports describe the same site and the same product substitution, they should be interpreted as complementary analyses of a single implementation rather than as independent replications. Ayhan and Yılmaz [33] observed lower consumable use with dry bed bathing but quantified neither emissions nor waste and conducted no life cycle assessment; this finding therefore indicates reduced material throughput rather than a demonstrated environmental benefit. Across the whole set, no study reported water use or energy consumption as separate environmental outcome metrics, and no study assessed environmental impact categories beyond global warming potential. Environmental outcome data were also absent from the pediatric and neonatal studies.

### 3.5. Economic Outcomes

Five studies reported economic or resource-use data [13,30,32,33,35]. The direction of effect depended on the product evaluated and on the costing perspective adopted. The life cycle assessment by Feely et al. [13] was the only analysis to report an overall cost increase: switching to reusable underpads and bed protectors increased total expenditure by approximately 3% (AUD 1005 over one year), with the increase primarily driven by reusable pinkies. The study also showed that environmental parity varied according to product type and the number of reuses achieved. Ayhan and Yılmaz [33] reported significantly lower consumable costs and a shorter procedure duration for dry bed bathing than for traditional bathing (*p* < 0.05). Tai et al. [35] found disposable-wipe baths to be shorter (23.8 versus 34.4 min; *p* < 0.01) and less costly overall (NTD 237.9 versus 255.3 per bath; *p* = 0.01), despite higher consumable costs, because laundry and nursing labour costs were lower. Larson et al. [32] reported a smaller difference in procedure duration (12.8 versus 14.4 min; *p* = 0.08) and lower mean total costs for disposable than traditional bathing (US$18.15 versus US$19.87 per bath); significantly fewer additional products were also used with disposable bathing (*p* < 0.001). In the neonatal setting, Babu et al. [30] estimated a diapering cost of Rs. 162 with disposable diapers compared with Rs. 241 with cloth diapers over an average six-day NICU stay, with the latter estimate incorporating purchase, stitching, and laundry costs. The comparability and transferability of these economic findings are limited by differences in currencies, study periods, local prices, labour costs, laundering arrangements, and the components included in each costing approach. Overall, the available studies suggest that economic performance is highly context- and product-dependent rather than consistently favouring either reusable or single-use systems.

### 3.6. Clinical Outcomes

Seven of the eight included studies reported clinical outcome data; only the life cycle assessment did not. The findings were grouped into three main sub-domains: pressure injury and skin integrity, microbiological and infection-related outcomes, and physiological responses to bathing. In the only study addressing pressure injury, Feely et al. [31] observed a lower incidence after the introduction of reusable linen (χ^2^(1) = 4.23; *p* = 0.040; Cramér’s V = 0.0447); however, linen type was no longer a significant predictor after multivariable adjustment. Significant predictors of pressure injury included APACHE III score (OR 1.011; 95% CI 1.003–1.019), age (OR 0.976; 95% CI 0.963–0.989), sex (OR 0.53; 95% CI 0.340–0.823), diabetes (OR 1.29; 95% CI 1.109–1.499), and ICU length of stay (OR 1.23; 95% CI 1.188–1.276). Nursing staff satisfaction with reusable linen was 87.2% [31].

Microbiological findings were not uniform in direction. Amir et al. [29] found a greater reduction in groin microbial counts with disposable prepackaged bathing than with traditional basin bathing on day 1 (*p* = 0.014) and again after crossover on day 2 (*p* = 0.033). These findings should be interpreted cautiously given the uncertainty surrounding the reported statistical approach. In contrast, Larson et al. [32], using paired observations in 40 patients, found no significant difference between disposable and traditional bathing in overall bath-quality scores or skin microbial counts.

With regard to physiological responses, Ayhan and Yılmaz [33] found that both dry and traditional bathing were associated with statistically significant changes over time in body temperature, blood pressure, heart rate, respiratory rate, and SpO_2_ (*p* < 0.05). Tai et al. [35] found no significant between-group differences in body surface temperature (*p* = 0.07), heart rate (*p* = 0.37), systolic blood pressure (*p* = 0.42), or oxygen saturation (*p* = 0.20). In the paediatric ICU, Öz et al. [34] reported that pulse and blood pressure were highest immediately after bathing and lowest at 30 min (*p* < 0.05); body temperature on day 2 was lower after traditional bathing, while SpO_2_ increased after both bathing methods and reached its highest values at 30 min. Overall, these studies indicate physiological changes associated with the bathing procedure itself, without consistent evidence of a clinically important advantage for either bathing modality.

Infection-related outcomes were reported in the neonatal study by Babu et al. [30]. Probable sepsis occurred in 101 of 253 neonates (39.9%) overall and was less frequent during the disposable-diaper period than during the cloth-diaper period (28% versus 45%; RR 0.75, 95% CI 0.62–0.92). After adjustment for birth weight, gestation, and sex, the odds of probable sepsis were 2.2-fold higher with cloth diapers (95% CI 1.26–3.99). Because this was a single-centre before–after study, the observed association should be interpreted in light of the potential for temporal and other uncontrolled confounding. The life cycle assessment by Feely et al. [13] did not assess clinical outcomes.

### 3.7. Synthesis of Evidence and Convergences Across Domains

The structured narrative synthesis identified substantial heterogeneity across study designs, settings, products, and outcome measures, precluding cumulative interpretation. Environmental findings favoured reusable linen for greenhouse gas emissions and landfill waste but were limited to two complementary reports from the same Australian ICU and one product family [13,31]. Economic findings were context- and product-dependent: disposable bathing systems and diapers were less costly in the settings examined by Ayhan and Yılmaz [33], Babu et al. [30], Larson et al. [32], and Tai et al. [35], whereas reusable linen was associated with an approximately 3% increase in overall expenditure [13]. Clinical findings were similarly inconsistent, with no uniform advantage of reusable or single-use products across pressure injury, microbiological, physiological, and infection-related outcomes. Overall, the main cross-domain gap was the absence of any single study evaluating environmental, economic, and clinical outcomes concurrently in the same population; convergence across domains therefore remains inferred across studies rather than demonstrated within an integrated evaluation.

## 4. Discussion

This systematic review is, to our knowledge, the first attempt to integrate clinical, economic, and environmental evidence on disposable and reusable products used in hygiene-related nursing care in intensive care. Eight studies met the eligibility criteria, a number that reflects the early stage of research in this field rather than a restrictive search strategy. The included studies were conducted in Australia, Turkey, Indonesia, Taiwan, the United States, and India. This distribution spans high- and middle-income contexts, but no study was conducted within a European Union healthcare system, where regulatory and procurement frameworks differ from those represented in the included evidence [36]. The absence of European evidence limits the transferability of the findings to those settings [37,38], since energy sources, waste-management systems, laundering infrastructure, and procurement regulation may all differ substantially from the contexts studied. No previous systematic review has examined the clinical, economic, and environmental dimensions of nursing hygiene practices in intensive care simultaneously, and the scarcity of available evidence amplifies the gap already identified by Gaetani et al. [9] and See [11].

That small evidence base is itself informative. The eight studies cluster into three product families of very unequal size: five compared a disposable or prepackaged bed bath with a traditional basin or soap-and-water bath, two examined reusables versus disposable linen and underpads in the same Australian ICU, and one compared disposable with reusable diapers in a neonatal unit. Only the bed-bathing group contains several studies addressing the same intervention with broadly comparable comparators, and it is therefore the only domain in which comparison across studies is meaningful; the environmental and neonatal findings each derive from a single centre. Research on sustainability in ICU hygiene remains organised around isolated products and local initiatives rather than forming a coherent body of comparative evidence. The primary studies also differ in population, design, comparator, and outcome measurement, so the synthesis is best understood as a set of heterogeneous signals rather than as a single effect estimate. This also explains why the environmental picture may appear clearer than the clinical and economic one: it rests on a single carefully modelled implementation, not on wider replication.

The hygiene products represented in this review, bed linen and underpads, prepackaged bathing systems, and neonatal diapers, cover only a fraction of the consumables used daily in intensive care. The absence of chlorhexidine-based oral hygiene products, antimicrobial-impregnated catheters, and perineal hygiene devices does not reflect their clinical irrelevance; it suggests instead that the literature has not yet addressed hygiene in the ICU as a unified practice area [39,40]. Research continues to address individual products in isolation, driven by infection-control priorities and episodic funding, rather than by systematic evaluation of the full hygiene-related consumable landscape [41,42,43].

On the environmental side, the available data are consistent with the broader literature on sustainability in intensive care. Gaetani et al. [9] estimated emissions of 88 to 178 kg CO_2_e per patient per day, identifying consumables and linen as major hotspots. Against this backdrop, the reduction from 7206 to 3605 kg CO_2_e documented by Feely et al. [13] falls within the range described in the life cycle assessment literature on healthcare textiles, where reductions of 30% to 93% have been reported depending on product type and local energy context [44]. Booth et al. [45] similarly found that reusable devices generally have a lower life cycle carbon footprint than single-use products, and McGain et al. [18] reached comparable conclusions in their overview of environmental sustainability in anaesthesia and critical care. Feely et al. [13] incorporated laundering within the life cycle system boundary, highlighting the importance of reprocessing infrastructure when evaluating reusable products, and the wider literature indicates that an inefficient or fossil-fuel-dependent laundry can offset a substantial part of the carbon savings obtained by switching to reusables [46,47,48]. The environmental benefit of reusable products therefore depends not only on the product chosen but also on the efficiency of the supporting infrastructure. This also delimits what the present review can support: only two of the eight included studies reported direct environmental outcomes, both from the same metropolitan ICU and the same product family, so our environmental finding indicates a plausible mechanism rather than an effect size that can be assumed to transfer to other settings.

The economic findings of this review do not point in a single direction, and that inconsistency is itself an important result. Compared by scope rather than by conclusion, the analyses become more intelligible: the four studies that costed consumables, procedure duration, and nursing labour favoured the single-use option, whereas the life cycle assessment, which also accounted for product acquisition across a reuse cycle, reported a modest overall increase of approximately 3% (AUD 1005 over one year). Neither reusable nor single-use products therefore demonstrated a consistent cost advantage across product categories [13,30,33], and much of the apparent disagreement between studies appears to reflect differences in the cost components counted and in the time horizon adopted rather than genuinely contradictory findings.

Economic performance appears highly context-dependent, shaped by product lifespan, institutional infrastructure, local prices and labour costs, and the clinical setting in which products are deployed. The analysis by Feely et al. [13] illustrates this logic: the case for reusables is not static but accumulates with use, and parity varies according to product type and the number of reuses actually achieved, so it is realised only where laundering is efficient and products are not prematurely discarded. This is consistent with the broader literature on healthcare procurement, which increasingly recognises that unit price alone is an inadequate basis for decision-making [25,39]. A practical implication follows: economic comparisons in this field should state explicitly which cost components and which time horizon they include, because analyses that omit reprocessing and replacement are not measuring the same quantity as those that include them.

On the clinical side, linen type was no longer a significant predictor of pressure injury after adjustment for APACHE III score, age, sex, diabetes, and ICU length of stay [31], which is consistent with evidence that patient characteristics rather than product type drive clinical risk [33]. This addresses a frequently cited barrier to adopting reusable products, although the claim the data support is the absence of an increased risk within a single before–after cohort rather than a demonstrated clinical benefit. The microbiological evidence is not uniform in direction: Amir et al. [29] found a greater reduction in groin microbial counts with disposable prepackaged bathing, whereas Larson et al. [32] found no significant difference in bath-quality scores or skin microbial counts. Because the two studies differed in sampling site, timing, and culture method, this discordance may reflect measurement heterogeneity as much as a true difference between products, and neither study measured a patient-level infection outcome. The physiological findings were not fully consistent across trials, and the methodological limitations identified reduce confidence in between-group causal inference. The available randomized evidence therefore does not establish a reproducible, clinically important advantage of either bathing modality. It is notable that 87.2% of nurses reported satisfaction with the reusable product after implementation [31], consistent with Lin et al. [49] on healthcare workers’ attitudes toward the sustainability of personal protective equipment.

The neonatal findings warrant separate consideration because they concern a product, a population, and an outcome represented by no other included study. Babu et al. [30] reported a lower incidence of probable sepsis during the disposable-diaper period, an association that persisted after adjustment for birth weight, gestation, and sex, but that arose from a single-centre before–after design in which temporal and other uncontrolled confounding cannot be excluded. Earlier literature on neonatal diapering points in a similar direction without resolving the question: Dore et al. [50] associated high-absorbency disposable diapers with improved skin protection and fewer sleep disruptions in neonates, while Wong et al. [51] reviewed the evidence on diaper dermatitis, skin integrity, and bacterial contamination and set it against the landfill burden generated by disposable products. Taken together, these sources suggest that neonatal diapering may be the one area in which single-use products carry a clinical advantage, and simultaneously the area in which the environmental cost of that advantage has never been quantified: neither the included studies nor the wider literature identified here provide a life cycle assessment of neonatal diapering in a critical care setting.

Concern over infection safety, rather than cost, remains the primary perceptual barrier to adopting reusables: in a survey of 465 healthcare workers, only 49% agreed that sterilized reusable PPE was as safe as single-use alternatives [49]. Available evidence suggests that structural factors, including staffing shortages, funding constraints, and procurement models, may represent greater barriers than healthcare workers’ willingness to adopt reusable systems [52].

**Figure 2:**
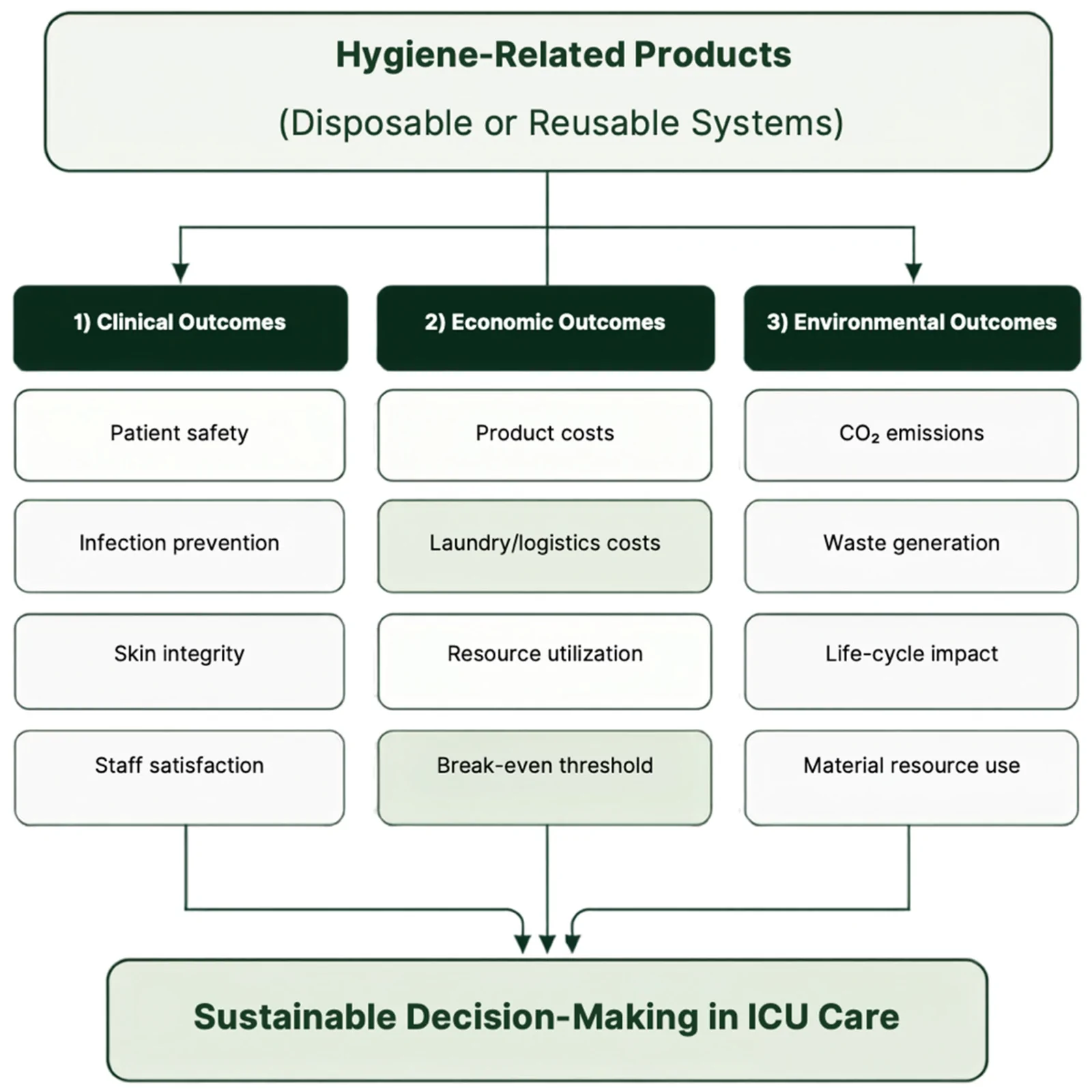
Integrated framework for evaluating hygiene-related products in intensive care. Clinical, economic, and environmental outcomes represent interconnected dimensions of value that should be considered simultaneously when evaluating disposable and reusable hygiene-related products in intensive care settings. The framework summarizes the integrated perspective emerging from the available evidence and highlights the need for multidimensional decision-making in sustainable ICU practice.

This review carries several implications for practice and policy. At the bedside, the available clinical and satisfaction data support carefully monitored local evaluations of reusable linen in adult ICUs, provided laundering standards and the number of reuses achieved are assessed prospectively; for bed bathing, procedure duration, cost, and staff preference may inform the choice between prepackaged and basin methods alongside clinical and infection-control considerations, since the physiological evidence does not consistently favour either modality. At the organisational level, procurement decisions would benefit from moving beyond unit price toward a total cost of ownership perspective incorporating life cycle carbon and waste. The persistent gap between evidence and perception argues for embedding sustainability literacy into critical care curricula. For research, the priority is a standardised outcome set covering clinical, economic, and environmental endpoints measured in the same population: no included study did so, which is why convergence across domains currently has to be inferred across studies rather than observed within any one of them. Comparative studies of this kind are costly and organisationally demanding, particularly where they require life cycle assessment methodology and laundry infrastructure tracking, and that difficulty itself helps explain the scarcity of available evidence [13,18]. Multicentre trials or prospective cohorts in European Union ICUs are needed most, because the available life cycle assessments reflect Australian and North American energy and laundering contexts that cannot be assumed to transfer. Figure 2 summarises the integrated perspective emerging from the available evidence.

### Limitations and Strengths

This review has several limitations. The eight included studies differed substantially in design, care setting, product type, and outcome measurement, which precluded quantitative meta-analysis and required a structured narrative synthesis. The evidence is also unevenly distributed: five studies addressed bed bathing, two addressed linen in the same Australian ICU, and one addressed neonatal diapering, so the environmental and neonatal findings each rest on a single centre. Two studies contributed the large majority of the pooled participant total, which means that the aggregate sample size reflects the size of two cohorts rather than the breadth of the evidence. Two of the four quasi-experimental studies used a before–after design, in which concurrent changes in staffing, case mix, or infection-prevention practice cannot be separated from the change in hygiene product. Blinding of those delivering the intervention was not achieved in the three randomized trials, while participant blinding was not achieved in two of them; allocation concealment was unclear or not achieved across the three trials. These limitations increase the risk of performance and detection bias and reduce confidence in the causal interpretation of between-group differences. In Öz et al. [34], the significant baseline age imbalance further limits the comparability of the randomized groups. The overall certainty of the evidence was not formally assessed using GRADE or an equivalent framework, so the conclusions should be interpreted with caution. Publication bias and selective outcome reporting remain possible and could not be assessed reliably across so small and heterogeneous a body of evidence.

The search was restricted to indexed, published literature in four databases. Although this improved consistency and reproducibility, it narrowed the scope of the review: grey literature was not searched, neither backward reference checking nor forward citation searching was undertaken, and relevant unpublished or non-indexed studies may therefore have been missed. Two reports could not be obtained and were classified as not retrieved, and three reports were excluded at the full-text stage because they were published in a language other than English or Italian; both are potential sources of bias that we cannot quantify. Because research on healthcare sustainability is published in many languages, eligible studies may have been missed, and the extent of this loss cannot be estimated. One included study concerned neonatal care and one concerned paediatric care, and neither is directly generalisable to adult ICUs, despite both meeting the prespecified eligibility criteria. Finally, no study was conducted within a European Union healthcare setting, which limits the direct transferability of the findings to those systems.

The review also has several strengths. To our knowledge, it is the first systematic review to examine the clinical, economic, and environmental dimensions of hygiene-related nursing care in intensive care within a single analytical framework. The search covered four major bibliographic databases, was rebuilt around PICOS concept blocks with database-specific controlled vocabulary and is reported as complete line-by-line histories so that it can be reproduced. Study selection, data extraction, and methodological appraisal were conducted independently by two reviewers, with blinded independent judgements applied during study selection and methodological appraisal and a third reviewer available to resolve disagreements. Appraisal instruments were matched to study design, with JBI checklists for the randomized and quasi-experimental studies and prespecified ISO 14040 and ISO 14044 criteria for the life cycle assessment, and item-level judgements are reported without conversion into percentage scores and without an arbitrary quality cut-off. The inclusion of adult, paediatric, and neonatal critical care settings across high- and middle-income countries broadened the perspective of the synthesis, and the structured narrative approach allowed evidence from distinct outcome domains to be considered together while preserving the differences between designs and settings.

## 5. Conclusions

The available evidence suggests that the answer differs by product rather than favouring one category of product overall. For bed linen and underpads in adult intensive care, the single implementation studied reported a substantial reduction in carbon emissions and landfill waste, no increase in pressure injury risk after adjustment, and high nursing acceptance, at a modest increase in overall expenditure. For bed bathing, prepackaged disposable systems were generally faster and, when costed from the perspective of the unit, less expensive, with physiological responses that did not differ consistently from traditional bathing, while the microbiological evidence remained discordant. For neonatal diapering, a single before–after study reported a lower incidence of probable sepsis and lower estimated costs with disposable diapers, a finding that has not been replicated and whose environmental counterpart has never been measured.

These findings support treating reusable systems as credible alternatives to single-use products in selected areas of adult intensive care, particularly for linen, while recognising that their value cannot be assumed to be uniform across products, settings, or laundering infrastructures. Because the economic and environmental results depend on which components each analysis counted, clinical outcomes, life cycle environmental impact, and the full cost of implementation should be considered together, and explicitly, when informing procurement and practice decisions.

The evidence base remains limited: eight heterogeneous studies, of which the environmental findings derive from a single Australian ICU and the neonatal findings from a single Indian unit, with no study conducted within a European Union healthcare setting, where energy sources, laundry systems, regulation, and procurement may differ substantially from those represented here. Comparative studies in European Union ICUs are therefore needed to establish which reusable products offer the greatest benefit and under which conditions. Above all, future research should measure clinical, economic, and environmental outcomes within the same study, so that sustainability decisions in critical care nursing can rest on integrated evidence rather than on inference across separate studies.

## Figures and Tables

**Table 1:** Search string.

(“Intensive Care Units”[Mesh] OR “Critical Care”[Mesh] OR “Critical Care Nursing”[Mesh] OR “intensive care”[tiab] OR “critical care”[tiab] OR ICU[tiab] OR ICUs[tiab] OR NICU*[tiab] OR PICU*[tiab] OR “critically ill”[tiab]) AND (“Hygiene”[Mesh] OR “Baths”[Mesh] OR “Oral Hygiene”[Mesh] OR “Toothbrushing”[Mesh] OR “Skin Care”[Mesh] OR “Bedding and Linens”[Mesh] OR “Incontinence Pads”[Mesh] OR “Diapers, Infant”[Mesh] OR hygiene[tiab] OR “bed bath”[tiab] OR “bed baths”[tiab] OR “bed bathing”[tiab] OR bathing[tiab] OR “body washing”[tiab] OR “perineal care”[tiab] OR “skin cleansing”[tiab] OR “oral care”[tiab] OR “mouth care”[tiab] OR toothbrush*[tiab] OR washcloth*[tiab] OR wipe[tiab] OR wipes[tiab] OR linen[tiab] OR linens[tiab] OR “bed sheet”[tiab] OR “bed sheets”[tiab] OR bedsheet*[tiab] OR “draw sheet”[tiab] OR “draw sheets”[tiab] OR underpad*[tiab] OR “incontinence pad”[tiab] OR “incontinence pads”[tiab] OR diaper*[tiab] OR nappy[tiab] OR nappies[tiab] OR “eye pad”[tiab] OR “eye pads”[tiab] OR towel*[tiab]) AND (“Disposable Equipment”[Mesh] OR “Equipment Reuse”[Mesh] OR disposable*[tiab] OR “single use”[tiab] OR “single-use”[tiab] OR reusable*[tiab] OR reuse[tiab] OR reused[tiab] OR washable[tiab] OR launder*[tiab] OR laundry[tiab])

Note: * Truncation wildcard used to retrieve word variants with different endings.

**Table 2:** JBI Critical Appraisal of Quasi-Experimental Studies.

Study	Q1	Q2	Q3	Q4	Q5	Q6	Q7	Q8	Q9
Amir et al., 2023 [29]	Y	Y	Y	Y	Y	Y	Y	Y	U
Feely et al., 2025 [31]	Y	Y	N	Y	N	Y	Y	Y	Y
Larson et al., 2004 [32]	Y	Y	Y	U	Y	Y	Y	N	Y
Babu et al., 2015 [30]	Y	Y	Y	Y	N	Y	Y	Y	Y

Legend: Q1 = Is it clear in the study what is the “cause” and what is the “effect” (i.e., there is no confusion about which variable comes first)? Q2 = Was there a control group? Q3 = Were participants included in any comparisons similar? Q4 = Were the participants included in any comparisons receiving similar treatment/care, other than the exposure or intervention of interest? Q5 = Were there multiple measurements of the outcome, both pre- and post-intervention/exposure? Q6 = Were the outcomes of participants included in any comparisons measured in the same way? Q7 = Were outcomes measured in a reliable way? Q8 = Was follow-up complete and, if not, were differences between groups in terms of their follow-up adequately described and analyzed? Q9 = Was appropriate statistical analysis used? Y = Yes; N = No; U = Unclear; NA = Not applicable. Methodological note. The revised JBI quasi-experimental checklist was used. Item-level judgements are reported without converting them into a numerical percentage score or applying an arbitrary quality cut-off.

**Table 3:** JBI Critical Appraisal of Randomized Controlled Trials.

Study	Q1	Q2	Q3	Q4	Q5	Q6	Q7	Q8	Q9	Q10	Q11	Q12	Q13
Tai et al., 2021 [35]	Y	U	Y	N	N	N	Y	Y	Y	Y	Y	Y	Y
Ayhan & Yılmaz, 2024 [33]	Y	U	Y	N	N	N	Y	Y	NA	Y	Y	Y	U
Öz et al., 2022 [34]	Y	N	N	Y	N	U	Y	Y	Y	Y	Y	Y	Y

Legend: Q1 = Was true randomization used for assignment of participants to treatment groups? Q2 = Was allocation to treatment groups concealed? Q3 = Were treatment groups similar at baseline? Q4 = Were participants blind to treatment assignment? Q5 = Were those delivering treatment blind to treatment assignment? Q6 = Were outcome assessors blind to treatment assignment? Q7 = Were treatment groups treated identically other than the intervention of interest? Q8 = Was follow-up complete and, if not, were differences between groups in terms of follow-up adequately described and analyzed? Q9 = Were participants analyzed in the groups to which they were randomized? Q10 = Were outcomes measured in the same way for treatment groups? Q11 = Were outcomes measured in a reliable way? Q12 = Was appropriate statistical analysis used? Q13 = Was the trial design appropriate, and were deviations from a standard parallel-group RCT accounted for in the conduct and analysis? Y = Yes; N = No; U = Unclear; NA = Not applicable. Interpretive note. For the randomized crossover trial, Q13 was used to capture crossover-specific safeguards, including sequence randomization, washout/carryover considerations, and the appropriateness of the analysis. Q9 was marked NA because allocation concerned treatment sequence rather than permanent parallel-group assignment.

**Table 4:** ISO 14040/14044- and Keil et al. [25]-Based Transparency Appraisal of the Life Cycle Assessment.

Item	Transparency Criterion	Rating	Appraisal Rationale
1	Aim clearly specified	Y	Objective explicitly compares environmental impact and cost of disposable versus reusable ICU linen.
2a	Functional unit specified	Y	Single patient use of a disposable or reusable bluey/pinky (and disposable incontinence underpants).
2b	Partial carbon footprint justified, if applicable	NA	A final product use is defined; this conditional item is not applicable.
3	Reference flow specified	Y	Product use counts and material flows required to deliver one patient use are defined and modelled.
4	Life-cycle stages described	Y	Raw materials, manufacture, transport, use/reuse, laundering/reprocessing, and end-of-life disposal are described.
5	Important unit processes reported	Y	Manufacturing, procurement transport, laundry transport, laundering, soiling, and landfill disposal are included.
6	Exclusions and reasons reported	Y	Laundry detergents are excluded with an estimate that their contribution is minor; other modelling exclusions/assumptions are stated.
7	System boundary specified	Y	The cradle-to-grave boundary is explicitly defined and illustrated.
8	Data sources reported	Y	Primary weighing/FT-IR data, manufacturer specifications, procurement data, supplier data, standards, and published emission sources are identified.
9	Temporal representativeness addressed	Y	The pre/post study periods and measurement periods are reported; contemporary standards and emission factors are identified.
10	Geographical representativeness addressed	Y	Manufacturing locations and transport routes to Melbourne are specified.
11	Technological representativeness addressed	Y	Product composition, expected reuse life, laundering practice, and site-specific use are described.
12	Data completeness assessed	N	No formal completeness/data-quality assessment or predefined completeness threshold is reported.
13	Carbon footprint expressed as CO_2_e	Y	Environmental impact is reported as kg CO_2_e.
14a	GHGs included are explicitly listed	N	The study applies IPCC GWP100 factors but does not explicitly list the individual greenhouse gases included.
14b	CO_2_-only analysis justified, if applicable	NA	The outcome is CO_2_e rather than CO_2_ alone.
15a	Characterisation factors specified	Y	IPCC 2021 GWP100 (100-year time horizon; version 1.03) is specified.
15b	CO_2_-only characterisation justified, if applicable	NA	Not applicable because CO_2_e is used.
16a	Allocation procedures reported	Y	Attributional LCA is stated; shared laundering processes are allocated proportionally to ICU use.
16b	Absence of allocation justified, if applicable	NA	Allocation is addressed; this conditional item is not applicable.
17	Outcomes reported per unit of analysis	Y	Per-use emissions are calculated; aggregate results and cost per 1000 ICU bed-days are also reported.
18	Carbon footprint reported by product/component	Y	Blueys, pinkies, and disposable incontinence underpants are reported separately.
19	Carbon footprint reported by life-cycle phase	Y	Manufacturing/disposal, transport, laundering/use contributions are reported, with further breakdown in Supplementary Table S1.
20	Influence of uncertainties/methodological choices discussed	Y	Reuse lifespan, landfill assumption, changing use patterns, soiling sample, and omitted detergents are explicitly discussed.
21	Quantitative sensitivity/scenario analysis performed	Y	Three reuse-life scenarios, counterfactual equivalent-use modelling, parity analysis, and ±10% cost sensitivity are reported.
22	Limitations critically discussed	Y	The authors discuss extrapolation, model misspecification, small soiling sample, pre/post non-equivalence, pricing confidentiality, omitted detergents, and carbon-only environmental scope.
Additional ISO 14040/14044 considerations			
Criterion		Rating	Rationale
Explicit conformity with ISO 14040/14044		Y	The Methods state that the analysis followed ISO life-cycle standards and cite ISO 14040 and ISO 14044.
Independent external critical review reported		N	No independent external critical review of the LCA is reported in the article.

Overall transparency assessment. High methodological transparency for the principal LCA domains. The study clearly reports its goal, functional unit, cradle-to-grave system boundary, inventory sources, allocation approach, IPCC characterisation method, product-level results, scenario/sensitivity analyses, and limitations. The main transparency limitations were the absence of a formal data-completeness/quality assessment, no explicit list of individual greenhouse gases contributing to CO_2_e, and no reported independent external critical review. Consistent with the review protocol, no percentage score or post hoc exclusion threshold was applied. Abbreviations and rating. Y = reported/adequately addressed; N = not reported or not adequately addressed; NA = not applicable; CO_2_e = carbon dioxide equivalent; GHG = greenhouse gas; ICU = intensive care unit; LCA = life cycle assessment. Appraisal basis. Criteria were informed by ISO 14040/14044 and the 22-item methodological transparency framework used by Keil et al. for comparative single-use versus reusable healthcare LCAs, which was derived from ISO standards and the carbon-footprint transparency catalogue of Lange et al.

**Table 5:** Characteristics of included studies and extracted data (structured narrative synthesis according to Popay et al. [28]).

Author/Year	Country	Study Design	Setting	Hygiene Material/Procedure	Comparator	Environmental Outcomes	Clinical Outcomes	Economic Outcomes	Main Findings
(Amir et al., 2023) [29]	Indonesia	Quasi-experimental two-group crossover study	Adult ICU	Disposable prepackaged bed bath using washcloths	Traditional basin bed bath	Not assessed	In 30 ICU patients, disposable bed bathing produced a greater reduction in groin microbial counts than traditional bathing on day 1 (*p* = 0.014) and after crossover on day 2 (*p* = 0.033).	Not assessed	Disposable bed bathing produced greater short-term reductions in groin microbial counts than traditional basin bathing in this crossover study.
(Ayhan & Yılmaz, 2024) [33]	Turkey	Randomized crossover clinical trial	General surgery ICU	Dry bed bath with single-use tissues	Traditional bed bath with soap and water	Lower consumable use with dry bathing; no formal environmental assessment	Both bath types produced statistically significant changes in body temperature, blood pressure, heart rate, respiratory rate, and SpO_2_ over time (*p* < 0.05), with no clinically relevant differences between bathing modalities.	Dry bed bathing had significantly lower consumable costs and a shorter procedure duration than traditional bathing (*p* < 0.05)	Dry bed bathing offered time and cost advantages while maintaining adequate hygiene and without clinically relevant differences in hemodynamic responses compared with traditional bathing.
(Babu et al., 2015) [30]	India	Prospective before–after study	Level II NICU	Disposable diapers	Reusable cloth diapers	Not assessed	Probable sepsis occurred in 101/253 neonates (39.9%) overall and was less frequent during the disposable-diaper period than during the cloth-diaper period (28% vs. 45%; RR 0.75, 95% CI 0.62–0.92). Adjusted odds of probable sepsis were 2.2-fold higher with cloth diapers (95% CI 1.26–3.99).	For an average 6-day NICU stay, estimated diapering cost was Rs. 162 with disposable diapers versus Rs. 241 with cloth diapers	Disposable diapers were associated with a lower incidence of probable neonatal sepsis and lower estimated diapering costs than reusable cloth diapers in this single-center before–after study.
(Feely et al., 2025) [31]	Australia	Quasi-experimental retrospective before–after study	Metropolitan ICU	Reusable linen and reusable underpads	Disposable linen and underpads (“blueys”)	Elimination of approximately 496 kg of landfill waste per year	Pressure injury incidence decreased after implementation (χ^2^(1) = 4.23; *p* = 0.040; Cramér’s V = 0.0447); however, linen type was not an independent predictor of pressure injury after multivariable adjustment.	No formal economic evaluation reported	After adjustment, linen type was not independently associated with pressure injury, and the transition to reusable linen eliminated approximately 496 kg of landfill waste annually. Nursing staff satisfaction with reusable linen was 87.2%.
(Feely et al., 2026) [13]	Australia	Process-based life cycle assessment (LCA)	Metropolitan ICU	Reusable underpads and bed protectors	Disposable underpads and bed protectors	50% reduction in carbon emissions (7206 vs. 3605 kg CO_2_e); approximately 3.6 tonnes of CO_2_e emissions and 2.2 tonnes of landfill waste avoided	No direct clinical outcomes assessed	Overall cost increase of approximately 3% (~AUD 1005)	Reusable linen substantially reduced carbon emissions and landfill waste, with a modest overall cost increase. The break-even point varied by product type and number of reuses.
(Larson et al., 2004) [32]	USA	Prospective crossover comparative study	Medical, surgical, and cardiothoracic ICUs	Prepackaged disposable bed bath	Traditional basin bed bath	Not assessed	Among 40 patients with paired observations, overall bath-quality scores and skin microbial counts did not differ meaningfully between methods. Site-specific microbiologic differences were small and considered unlikely to be clinically significant.	Mean bath time was 12.8 min with disposable versus 14.4 min with traditional bathing (*p* = 0.08). Mean total cost was US$18.15 versus US$19.87 per bath, respectively. Fewer additional products were used with disposable bathing (*p* < 0.001).	Disposable bathing required fewer products, had slightly lower overall costs, and was strongly preferred by nurses (77.5% overall preference; *p* < 0.001), with no meaningful disadvantage in bath quality or skin microbiology.
(Öz et al., 2022) [34]	Turkey	Randomized controlled trial	Pediatric ICU	Disposable bed bath using prepackaged body-cleansing wipes	Traditional bed bath using soap and water	Not assessed	Among 68 children (34 per group), pulse and blood pressure were highest immediately after bathing and lowest at 30 min (*p* < 0.05). On day 2, body temperature was lower after traditional bathing; SpO_2_ increased after both methods and was highest at 30 min.	Not assessed	Both bathing methods were associated with acceptable physiologic responses in hemodynamically stable PICU patients; differences were mainly observed in post-bath temperature and SpO_2_ patterns.
(Tai et al., 2021) [35]	Taiwan	Randomized controlled experimental study	Medical ICU	Bed bath using disposable wipes	Traditional soap-and-waterbed bath	Not formally assessed	No significant between-group differences were observed in body surface temperature (*p* = 0.07), heart rate (*p* = 0.37), systolic blood pressure (*p* = 0.42), or oxygen saturation (*p* = 0.20).	Disposable-wipe baths were shorter (23.8 vs. 34.4 min; *p* < 0.01) and had lower total cost (NTD 237.9 vs. 255.3 per bath; *p* = 0.01), despite higher consumable costs. Laundry and nursing labor costs were lower.	Disposable-wipe bathing reduced procedure time and total cost without adversely affecting vital-sign trends. Staff generally favored disposable wipes for convenience and time saving.

Note. ICU = intensive care unit; NICU = neonatal intensive care unit; LCA = Life Cycle Assessment; RCT = randomized controlled trial; NA = not available/not assessed; CO_2_e = CO_2_-equivalents; AUD = Australian dollar; Rs. = Indian rupee; LOS = length of stay.

## Data Availability

The original contributions presented in this study are included in the article. Further inquiries can be directed to the corresponding author.

## Supplementary Materials

The following supporting information can be downloaded at: https://www.mdpi.com/article/10.3390/nursrep16090335/s1, Table S1: Complete electronic database search strategies, search dates, and records retrieved.

## Public Involvement Statement

No public involvement in any aspect of this research.

## Guidelines and Standards Statement

This manuscript was drafted in accordance with the Preferred Reporting Items for Systematic Reviews and Meta-Analyses (PRISMA) 2020 statement [19].
