# Supplementary material for: Beyond Disposability: A Systematic Review of Reusable and Single-Use Products in Hygiene-Related Nursing Care in the ICU
Source: Nurs Rep. 2026 Sep 13;16(9):335. doi: 10.3390/nursrep16090335 (PMC13610850; doi:10.3390/nursrep16090335)
Supplement: Supplementary file 1 [file nursrep-16-00335-s001.zip › nursrep-4468484-supplementary.pdf]

Supplementary Materials

Beyond Disposability: A Systematic Review of Reusable and Single-Use Products in Hygiene-Related Nursing Care in the ICU

Table S1. Complete electronic database search strategies, search dates, and records retrieved.

Database	Date searched	Search strategy	Records retrieved
MEDLINE via PubMed	11 February 2026	((((((((((((((critical care[Title/Abstract]) OR (intensive care units[Title/Abstract])) OR (ICU[Title/Abstract])) AND (body hygiene[Title/Abstract])) AND (soap water[Title/Abstract])) OR (incontinence pad[Title/Abstract])) OR (bed sheet[Title/Abstract])) OR (diaper[Title/Abstract])) OR (eye pad[Title/Abstract])) OR (toothbrush[Title/Abstract])) AND (wet wipes[Title/Abstract]) AND (nursing[Title/Abstract])) AND (disposable[Title/Abstract])) OR (single use[Title/Abstract])) OR (reusable[Title/Abstract])) AND (environment*[Title/Abstract])) AND (sustainab*[Title/Abstract])	*892*
Scopus	11 February 2026	TITLE-ABS-KEY (critical AND care OR intensive AND care AND unit OR icu) AND TITLE-ABS-KEY (body hygiene OR soap water OR incontinence pad OR bed sheet OR diaper OR eye pad OR toothbrush OR wet wipes) AND TITLE-ABS-KEY (disposable OR single AND use OR reusable) AND TITLE-ABS-KEY (sustainab* OR environment*)	*7*
Web of Science Core Collection	11 February 2026	critical care OR intensive care unit OR ICU (Abstract) and body hygiene OR soap water OR incontinence pad OR bed sheet OR diaper OR eye pad OR toothbrush OR wet wipes (Abstract) and disposable OR single use OR reusable (Abstract) and sustainab* OR environment* (Abstract)	*174*
CINAHL Complete (EBSCOhost)	11 February 2026	XB (critical care OR intensive care units OR ICU) AND XB (body hygiene OR soap water OR incontinence pad OR bed sheet OR diaper OR eye pad OR toothbrush OR wet wipes) AND XB (disposable OR single use OR reusable) AND XB (environment* OR sustainab*)	*3*

Note. All searches were conducted on 11 February 2026 without publication-date restrictions. A total of 1,076 records were retrieved before duplicate removal: MEDLINE via PubMed (n = 892), Scopus (n = 7), Web of Science Core Collection (n = 174), and CINAHL Complete via EBSCOhost (n = 3).
